# Iliac Arteriovenous Fistula and Pseudoaneurysm Secondary to Gunshot Trauma

**DOI:** 10.3390/diagnostics15151882

**Published:** 2025-07-27

**Authors:** Ibrahim Akbudak, Muhammed Tekinhatun, Mehmet Sait Duyu, Fatih Cihan

**Affiliations:** Department of Radiology, Dicle University, 21280 Diyarbakir, Turkey; mtekinhatun@gmail.com (M.T.); mehmetsait295@gmail.com (M.S.D.); htf.nhc@gmail.com (F.C.)

**Keywords:** trauma, vascular injury, arteriovenous fistula, pseudoaneurysm, gunshot wound

## Abstract

Abdominal arteriovenous fistula [AVF] is a rare but serious complication of penetrating trauma, often associated with high morbidity and mortality. This report presents the case of a 24-year-old male who sustained multiple gunshot wounds, leading to the formation of an ilio-iliac AVF and a pseudoaneurysm. The patient arrived at the emergency department hemodynamically unstable, with bullet wounds to the forearm, thigh, and lumbosacral region. Initial non-arterial phase CT revealed a pseudoaneurysm anterior to the right external iliac vessels and a surrounding hematoma, raising suspicion for AVF. A second biphasic CTA confirmed an AVF connection between the right external iliac artery and external iliac vein, as well as the arterialization of the vein. Additionally, fat stranding and bowel wall thickening suggested potential hollow viscus injury. Due to the patient’s unstable condition and possible intra-abdominal injuries, an open laparotomy was performed. A stent was placed in the right external iliac artery, the vein was primarily repaired, and serosal injuries to the duodenum and cecum were surgically addressed. The patient recovered gradually, although a persistent serous discharge was noted and managed in follow-up. This case highlights the importance of considering AVF in penetrating abdominal trauma and the critical role of biphasic CTA in diagnosis and surgical planning.

**Figure 1:**
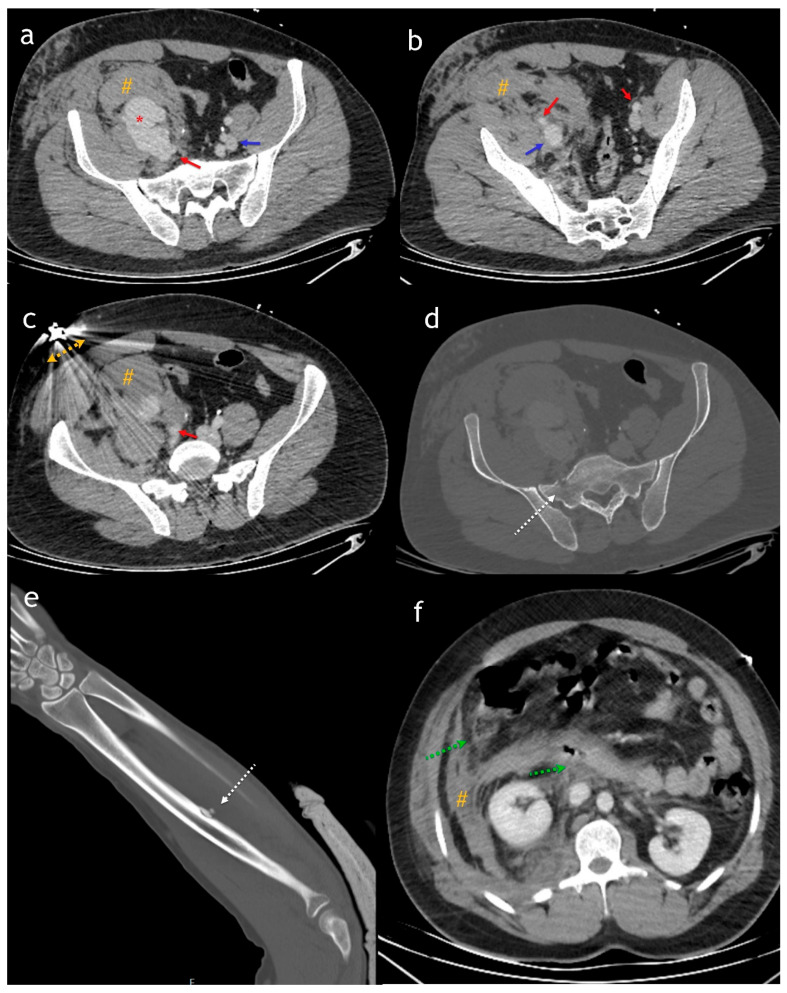
Axial (**a**–**c**) images from an abdominal CT scan obtained in the portal phase [soft tissue window] show the iliac artery [red arrow], iliac vein [blue arrow], a large hematoma [#], and a suspected pseudoaneurysm [*]. However, due to the acquisition phase, both iliac veins appear similar, making the AVF undetectable. In the bone window (**d**), a secondary fracture [dashed white arrow] is observed in the sacrum and left radius (**e**), caused by the bullet’s trajectory. In the upper abdominal sections (**f**), more pronounced fat stranding around the duodenum and increased wall thickness in the duodenum and cecum [dashed green arrow] and hematoma [#] raise suspicion of hollow viscus injury. Arterial phase CTA is the most effective diagnostic modality for AVF, as it demonstrates an enhancing venous lumen with the same density as the accompanying artery, significantly more than other veins [1,2]. Additionally, the direct connection of a pseudoaneurysm with both the injured artery and vein can be better visualized. The venous phase is also useful, as it may reveal solid organ injuries. If contrast media extravasation is present, indicating active hemorrhage, the volume of extraluminal contrast increases in the venous phase [1,2]. Gunshot wounds to the abdomen carry a high risk of vascular injury, and high-quality imaging is essential for accurate diagnosis. Biphasic computed tomography angiography (CTA), which includes arterial and portal venous phases, is the gold standard for evaluating both vascular structures and solid organ injuries [3,4]. The arterial phase is particularly crucial for detecting arterial injuries, pseudoaneurysms, and active contrast extravasation, while the portal venous phase enhances the visualization of solid organ injuries, venous abnormalities, and secondary findings such as bowel wall thickening or hematomas. Proper timing in imaging is crucial, as in this case, an initial miscalculation led to diagnostic difficulties.

**Figure 2:**
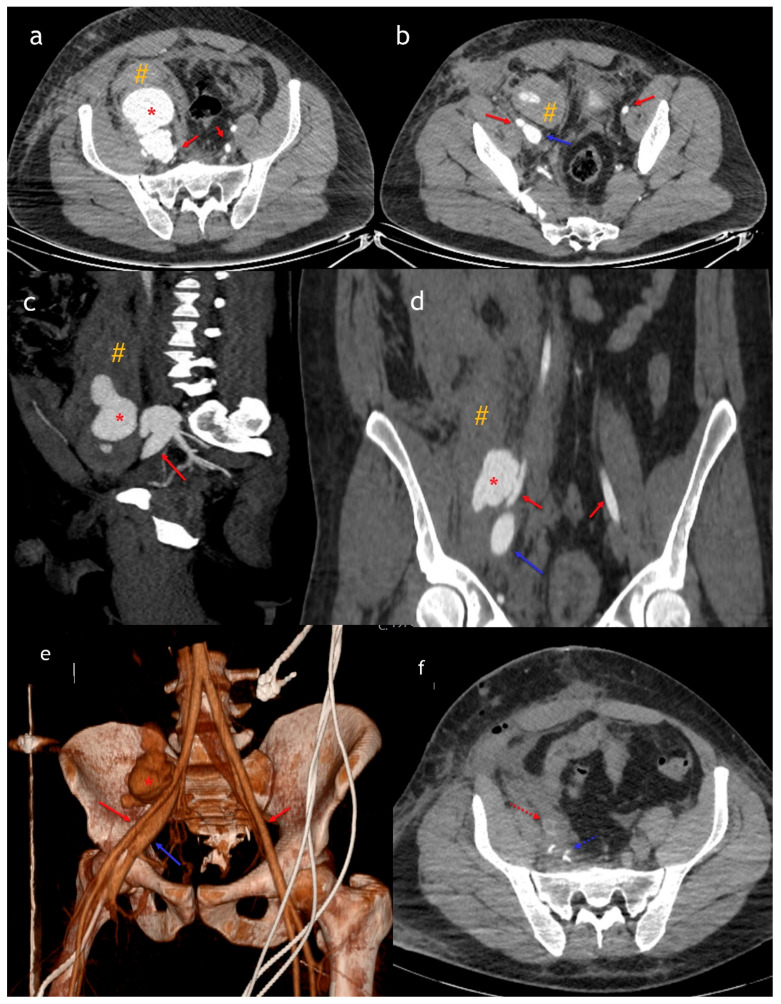
Axial (**a**,**b**), sagittal (**c**), coronal (**d**), and 3D (**e**) arterial phase CT images show a fistula and pseudoaneurysm [*] between the iliac artery [red arrow] and iliac vein [blue arrow], along with an accompanying hematoma. Postoperative imaging (**f**) reveals a stent placed in the iliac artery [red dashed arrow] and metallic clips at the level of the iliac vein [dashed blue arrow]. The patient underwent a second CT scan with arterial phase imaging a few hours later, which revealed a 6 cm diameter pseudoaneurysm connected to both the external iliac artery and vein. The inferior vena cava lumen demonstrated arterialization, while veins distal to the central venous catheter in the femoral vein showed normal enhancement. At our center, biphasic CTA [including both arterial and venous phases] is the preferred imaging modality when vascular injury is suspected. Abnormal communications between arteries and veins are defined as arteriovenous fistulas [AVFs] [5]. A second biphasic CTA confirmed an AVF between the right external iliac artery and vein, along with the arterialization of the vein. This phenomenon occurs due to the direct shunting of high-pressure arterial blood into the venous system, resulting in early and intense venous enhancement that mimics arterial opacification—a key imaging clue in the diagnosis of AVFs [6]. These can occur secondary to aneurysmal erosion or interventional procedures such as biopsies, surgery, or trauma. Compared to the craniocervical region, abdominal AVFs are relatively rare [1,5]. They can develop secondary to lumbar disk surgery or aneurysmal erosion—which may be associated with atherosclerosis, Ehlers–Danlos syndrome, Marfan syndrome, neoplastic invasion, or mycotic and syphilitic aneurysms—or penetrating trauma [6]. Trauma is a rare cause of AVF, with an incidence ranging from 2.3% to 3.9% [7,8]. Endovascular therapy is generally a less invasive and safer treatment option in hemodynamically stable patients, whereas open surgery remains the first-line treatment for unstable trauma patients, as was the case here [6]. The recent literature also highlights the importance of endovascular techniques in managing arteriovenous fistulas and related complications, as demonstrated by Ascoli Marchetti et al. in their case report on aortocaval fistula treatment [6]. Given the rarity of abdominal AVFs, hematomas, and pseudoaneurysms following gunshot wounds, accurate diagnosis and prompt treatment are essential for survival. This study aims to highlight the diagnostic challenges and treatment strategies in managing such abdominal vascular injuries.

## Data Availability

Not applicable.
